# The world is random: a cognitive perspective on perceived disorder

**DOI:** 10.3389/fpsyg.2014.00606

**Published:** 2014-06-13

**Authors:** Hiroki P. Kotabe

**Affiliations:** Center for Decision Research and Department of Psychology, University of ChicagoChicago, IL, USA

**Keywords:** perceived disorder, physical disorder, social disorder, environmental disorder, broken windows

## Abstract

Research on the consequences of perceiving disorder is largely sociological and concerns broken windows theory, which states that signs of social disorder cause further social disorder. The predominant psychological explanations for this phenomenon are primarily social. In contrast, I propose a parsimonious cognitive model (“world-is-random” model; WIR) that may partly account for these effects. Basically, WIR proposes that perceiving disorder primes randomness-related concepts, which results in a reduction to one’s sense of personal control, which has diverse affective, judgmental, and behavioral consequences. I review recent developments on the psychological consequences of perceiving disorder and argue that WIR can explain all of these findings. I also cover select correlational findings from the sociological literature and explain how WIR can at least partly explain them. In a general discussion, I consider possible alternative psychological models and argue that they do not adequately explain the most recent psychological research on disorder. I then propose future directions which include determining whether perceiving disorder causes a “unique psychology” and delimiting boundary conditions.

Most of the research on the possible effects of perceived disorder on humans is sociological and concerns broken windows theory (BWT). BWT basically states that signs of social disorder (e.g., broken windows) cause further social disorder (e.g., more vandalism, theft; Wilson and Kelling, 1982; see also Keizer et al., 2008). Explanations for broken windows effects (BWE) are generally social. They focus on social norms, social signaling, and lack of social monitoring. In contrast, in this article, I propose a cognitive, “inside-one-head” model of the psychological consequences of perceiving disorder. After proceeding with the cognitive analysis, I turn back to the important naturally occurring social phenomena that I believe are partly explained by this cognitive model.

Before reviewing some recent developments relevant to this model, I should operationalize what I mean by “perceived disorder” (and “perceived order”): Perceived disorder is an interpreted state of the world in which things are in non-patterned and non-coherent positions. Oppositely, perceived order is an interpreted state of the world in which things are in patterned and coherent positions. Note that these broad definitions include all animate or inanimate things (i.e., all things that can be represented in mental “chunks”), and thus may apply both to purely physical disorder (e.g., objects randomly scattered about on a computer screen) and social disorder (e.g., littering, crime). The key requirement is that the stimuli are processed as non-patterned and non-coherent chunks.

There seems to be a developing interest among psychologists in the consequences of perceived disorder on human psychology (not necessarily in the context of BWT, however). Recently, some consequences of perceived disorder pertinent to the proposed model were documented by – in chronological order – Heintzelman et al. (2013), Vohs et al. (2013), and Chae and Zhu (2014): Heintzelman et al. (2013) documented a psychological state consequence of disorder. Across four studies, they manipulated perceived disorder either by (a) presenting people with pictures of seasons in temporal sequence (e.g., autumn, winter, spring, summer) or random sequence (e.g., winter, autumn, summer, spring; Experiments 1 and 2) or, in a more stripped-down presentation, (b) presenting people with semantic triads (i.e., Remote Associates Test items; Mednick, 1962) that were either coherent (e.g., “falling, actor, dust”; common associate: star) or incoherent (e.g., “belt, deal, nose”; Experiments 3 and 4). Subsequently, people across all four experiments reported less meaning in life in the disorderly condition than in the orderly condition (*d*s ranging from 0.37 to 0.54). Vohs et al. (2013) documented some judgment and behavioral consequences of perceived disorder. Across three experiments, they manipulated the immediate lab environment to be either orderly or disorderly. People in disorderly environments donated less (*d* = 0.73) and chose fewer healthier snacks (φ = 0.37; Experiment 1); they were rated as more creative in coming up with alternative uses for an ordinary object (*d* = 0.61; Experiment 2); and they showed stronger preference for an unconventional product whereas those in the orderly environment showed stronger preference for a conventional product (interaction, φ = 0.20; Experiment 3). Most recently, Chae and Zhu (2014) documented some other judgment, behavioral, and state consequences of perceived disorder. Across four experiments, they manipulated perceived disorder à la Vohs et al. (2013) – by having people do tasks in either a disorderly or orderly lab environment. Compared with people in the orderly environment, people in disorderly environments reported being willing to pay more for tempting but unnecessary products (*d* = 0.43; Experiment 1); they reacted slower in a Stroop task (*d* = 0.46) and reported feeling more depleted (*d* = 0.69; Experiment 2); and they did not persist as long on an unsolvable puzzle (*d* = 0.42, Experiment 3; *d* = 0.73, Experiment 4). Further, and most germane to the proposed model, they found in Experiment 4 that a threat to feeling in control mediated the effects of perceived disorder on persistence.

Perceived disorder apparently has a variety of psychological consequences for affect (broadly defined, see Gross and Thompson, 2007), judgment, and behavior. Is there a common process underlying these effects? Next, I will elaborate on a model that could account for the foregoing experimental findings as well as correlational findings in the sociological literature. In a general discussion, I will discuss three possible alternative psychological models that may explain some but not all of these findings, as well as future directions.

## THE WORLD IS RANDOM

**FIGURE 1:**
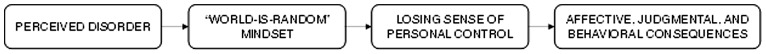
**The world-is-random (WIR) model**.

To follow along, see **Figure 1** for a diagram of the proposed world-is-random model (WIR): Neglecting randomness, chance, and luck leads us to an illusion of control. WIR proposes that perceiving disorder primes concepts related with randomness/chance/luck (thus creating a “world-is-random” mindset). It may thus lead us to (accurately) believe we have less control over outcomes in low-control/high-chance situations because we weight available representations related to randomness/chance/luck more (Tversky and Kahneman, 1973). Through the same mechanism, it may even lead us to (erroneously) believe we have less control over ourselves when strongly tempted (i.e., when in a state of low-control/high-chance). This sense of losing personal control may have a variety of affective, judgmental, and behavioral consequences.

WIR can account for the experimental findings discussed earlier. Regarding the investigation on perceived disorder and meaning in life by Heintzelman et al. (2013), WIR explains these results as a negative consequence of losing a sense of personal control. Feeling in control is a fundamental human need (White, 1959; Bandura, 1977; Deci and Ryan, 1985; Higgins, 2011). If not met, humans suffer. One plausible manifestation, according to self-determination theory, is a feeling that life is meaningless because one cannot control outcomes (unfulfilled competence need) or choose their own way (unfulfilled autonomy need).

The sense of losing control resulting from perceiving disorder can also explain the results from the experiments by Vohs et al. (2013). In Experiment 1, people in a disorderly environment (a) donated less and (b) chose fewer healthy snacks. Having personal control means being able to agentically influence outcomes (White, 1959; Deci and Ryan, 1985). Thus, people whose sense of personal control is reduced, by definition, see their actions (e.g., donating) as having less consequence. Similarly, people whose sense of personal control is reduced are likely to see their efforts to control oneself as more in vain, thus it follows that they would exert less self-control. In Experiment 2, people in a disorderly environment were rated as more creative. Research has shown that people are more creative when they enter a state of “flow,” which necessitates, among other operating conditions, a reduction in executive control (Csikszentmihalyi, 1997). WIR proposes that, through priming and increasing the judgment weight of randomness-related concepts, perceived disorder decreases our sense of control over oneself. Such changes to our beliefs may reduce the motivation to exert executive control (Job et al., 2010; Kotabe and Hofmann, submitted), facilitating advancement into a flow state of unshackled creativity. Regarding Experiment 3, people in a disorderly environment more strongly preferred an unconventional product whereas people in an orderly environment more strongly preferred a conventional product. WIR explains these results similar to how it explains the results from Experiment 2. By reducing our sense of personal control and use of control resources, perceived disorder may facilitate a state of flow in which conventional boundaries “disappear.”

World-is-random explains the results from the experiments by Chae and Zhu (2014) in a slightly different way. It assumes that the sense of losing control is threatening, and that this threat, in turn, is depleting to cognitive resources (Glass et al., 1969; Baumeister et al., 2007; Inzlicht and Kang, 2010), thus resulting in more impulsive behaviors across various domains. Accordingly, in Experiment 1, people in a disorderly environment were willing to pay more for tempting products and, in Experiment 2, people in a disorderly environment were slower to react in a Stroop task and reported feeling more depleted. In Experiments 3 and 4, people in a disorderly environment persisted less on an unsolvable puzzle. Moreover, the authors showed that a reduction in and threat to one’s sense of personal control mediated the effect of perceived disorder on persistence in Experiment 4, consistent with the mechanisms I propose.

WIR can also (partly) explain a variety of correlational findings in the sociological literature. For brevity, and because this paper does not focus on the sociological consequences of perceived disorder, I will only review select research intended to demonstrate the breadth of findings WIR may at least partly account for (for a summary, see **Table 1**)^1^. First, take a cross-sectional study by Geis and Ross (1998). Analyzing data of a representative sample of 2,482 adults, aged 18–92 years, in Illinois (from the 1995 survey of Community Crime and Health), they found that neighborhood-level disorder was associated with perceived powerlessness. WIR can explain this similarly to how it explains the “meaning in life” findings by Heintzelman et al. (2013). That is, by making the world feel random, people start to lose a sense of control which manifests itself in negative outlooks on life such as feeling powerless and meaningless. Another likely manifestation is distress; Cutrona et al. (2000) found that neighborhood-level disorder was associated with distress, and this was moderated by life outlook, temperament, and quality of relationships. Specifically, disorder was associated with higher distress among people with a more negative life outlook, more negative temperament, and low-quality relationships. Importantly, this study suggests that although perceiving disorder may result in negative affect via a reduction in a sense of personal control, it is not inevitable. This is consistent with recent psychological research showing that people sometimes buffer against the threat of losing control through compensatory control mechanisms (Whitson and Galinsky, 2008; Kay et al., 2010). It seems to be currently assumed that people generally possess and use this ability, but future research may find that there are individual differences. This would clearly have implications for the proposed model, and may necessitate including moderators. Ross (2000) analyzed other data from the 1995 survey of Community Crime and Health and found that neighborhood disorder was associated with self-reported depression. Again, these results are consistent with the idea that perceived disorder results in a sense of losing control, which has insidious psychological consequences. Lastly, Perkins and Taylor (1996) surveyed 412 people across 50 neighborhoods in Baltimore to evaluate the relationship between neighborhood disorder and fear of crime. Three methods were used to measure both physical and social dimensions of neighborhood disorder: self-reported resident perceptions, on-site observations by trained raters, and newspaper content analysis. All three measures of neighborhood disorder predicted fear of crime, corroborating the general definition of perceived disorder assumed in WIR. As fear is an affective response to threat (Watson, 2000), these findings can be explained by the sense of threat resulting from losing a sense of control: When we are threatened, we generate a primitive fight-or-flight response in which we pay particular attention to sources of threat in our environment (so we can avoid them or prepare for them), such as lurking criminals. It follows that people would start to lose a sense of security and safety, as documented in this study.

**Table 1:** Select experimental and correlational findings on the psychological consequences of disorder.

Method	Reference	Finding
Experimental	Cialdini et al. (1990), Cialdini (2007)	Perceived disorder in one domain increases the creation of disorder in the same domain
	Keizer et al. (2008)	Perceived disorder in one domain (e.g., graffiti) spreads to creation of disorder in another domain (e.g., littering)
	Keizer et al. (2008)	Perceived disorder weakens “act appropriately” goal and consequently strengthens hedonic (e.g., litter) and gain (e.g., steal) goals
	Heintzelman et al. (2013)	Perceived disorder decreases self-reported meaning in life
	Vohs et al. (2013)	Perceived disorder decreases healthy choices and generosity
	Vohs et al. (2013)	Perceived disorder decreases conventionality and increases creativity
	Chae and Zhu (2014)	Perceived disorder increases self-regulatory failure
Correlational	Geis and Ross (1998)	Neighborhood disorder associated with perceived powerlessness
	Cutrona et al. (2000), Linares et al. (2001)	Neighborhood disorder associated with psychological distress
	Ross (2000)	Neighborhood disorder associated with depression
	Perkins and Taylor (1996)	Neighborhood disorder associated with increased fear of crime and decreased sense of safety

## GENERAL DISCUSSION

In the following discussion, I consider possible alternatives to WIR and explain why they may be inadequate. I then discuss some future directions for psychological research on perceived disorder.

## ALTERNATIVE EXPLANATIONS

### COGNITIVE DISFLUENCY EXPLANATION

Perceived disorder might be cognitively processed more disfluently than perceived order. Disfluency is thought to make people think more deeply and abstractly (Alter, 2013). Therefore, perceived disorder might have effects on judgment and behavior through disfluency, though it is unlikely that disfluency would have as severely negative affective consequences as the sense of losing control proposed by WIR. That said, both accounts could explain how perceived disorder may result in more accurate judgments in low-control/high-chance situations – the difference being the mechanism through which this happens. WIR would make this prediction by stating that disorder in the environment results in priming concepts related to randomness/chance/luck, and thus, through the availability heuristic, these concepts are appropriately weighted more in judgment. The cognitive fluency explanation would make this prediction by stating that people make more accurate judgments in a disorderly environment because they think harder (utilizing more effortful “system 2” processing, Kahneman, 2011). Both mechanisms could jointly work together, however, the recent experimental research reviewed in this article is more consistent with the conditioning/priming account of WIR than a disfluency account, since it seems unlikely that cognitive disfluency would result in the sense of losing control. If anything, it would result in the opposite.

### SOCIAL/RATIONAL AGENT EXPLANATION

This general and prevalent view concerns how perceived disorder may signal information about social norms and social monitoring. It suggests that people’s judgments and behaviors in disorderly environments can be understood as rationally aimed at minimizing expected costs and maximizing expected benefits, given the available social information. Regarding social norms and signaling, environmental disorder (e.g., litter) defines the descriptive norm (“littering is okay here”) which inhibits the effectiveness of the injunctive norm (e.g., no littering policy), thus the perceived costs of littering are lowered and people litter (Cialdini et al., 1990; Cialdini, 2007). Regarding social monitoring, perceived disorder may reduce the perceived costs of crime (e.g., littering) by signaling that monitoring/policing is low and thus punishment is unlikely, thus reducing expected costs of committing crimes. While these explanations can account widely for BWE (thus their popularity), they do not provide a clear account for the recent advances in psychological research on perceived disorder, which has documented that perceived disorder results in threats to and reductions of the sense of control. That being said, I do not doubt that perceived disorder can have such social effects, which is partly why I think that there may be a “unique psychology” (i.e., a distinct constellation of psychological phenomena) caused by perceiving disorder―more on that later.

### GOAL-BASED EXPLANATION

This explanation makes similar predictions to the social/rational agent explanation. Basically, a reduction in the expected costs of a crime (e.g., littering) due to perceived disorder of that form in the environment results in a weakened ‘act-appropriately’ goal and consequently increases the strength of “hedonic” (e.g., littering) and “gain” (e.g., stealing) goals (see Lindenberg and Steg, 2007; Keizer et al., 2008). Thus, people who see litter in the environment also commit other crimes such as illegally using graffiti and stealing. Again, while this can explain BWE and the “spreading of disorder” (see Keizer et al., 2008), it does not seem to relate with the documented reduction in a sense of personal control.

## FUTURE DIRECTIONS

### A UNIQUE PSYCHOLOGY?

As mentioned above, one direction for future research is to determine whether there is a distinct cluster of psychological consequences caused by perceiving disorder. Is there more to it than just priming randomness-related concepts and the associated consequences proposed by WIR? To my knowledge, there is no experimental evidence yet to confirm this. Although Keizer et al. (2008) proposed that goals are activated and deactivated in response to perceiving disorder, they did not measure this, and rather it is implied from the behavioral evidence which may be completely accounted for by reduced self-control. However, given the related research on social norms and signaling, I do not doubt that there is indeed more to the story. Further research can determine this conclusively.

### INDIVIDUAL DIFFERENCES?

Cutrona et al. (2000) provides correlational evidence that effects of perceiving disorder may be moderated by individual differences such as negativity and poor relationships. Moving forward, we should experimentally test whether personality measures of negative temperament (e.g., adult temperament questionnaire, Rothbart et al., 2000) and relationship quality (e.g., the positive relations with others scale, Ryff, 1989) have moderating effects and why. One possibility is that some people may not use compensatory control mechanisms (effectively). Further, Vohs et al. (2013) assumes that individual differences in reactions to perceiving disorder may translate into reactions to situational-level disorder. In light of this proposition, it may make sense to test whether there are interactions between classic personality measures regarding reactions to perceived disorder – such as preference for consistency (Cialdini et al., 1995), need for structure (Neuberg and Newsom, 1993), need for closure (Webster and Kruglanski, 1994), and ambiguity tolerance (Norton, 1975) – and perceiving disorder in one’s surroundings.

### DEPENDENT VARIABLES

To advance an interdisciplinary connection between the psychology of perceived disorder and the sociology of BWT, it will be important to develop laboratory measures analogous to those interpreted in the sociology of BWT. What would a laboratory analog be for “throwing a rock through the window of an abandoned building?” One may look to the aggression literature for inspiration. For example, research in this domain has employed creative behavioral measures such as serving hot sauce to a confederate (Bushman et al., 2005), blasting a confederate with aversive noise (Bushman et al., 2005), and delivering ostensibly painful shocks (Zillmann, 1971) to assess aggressive tendencies, which may have some parallels with criminal behaviors such as vandalism and theft.

## CONCLUDING REMARKS

Research on the psychology of perceived disorder is a new and exciting development. In this article, I propose a parsimonious cognitive model possibly explaining a variety of effects and relationships concerning perceived disorder documented across the psychological and sociological literatures. To recap, WIR proposes that perceiving disorder results in a threatening sense of losing personal control (via priming randomness-related concepts), which can account for a variety of affective, judgmental, and behavioral findings in the literatures. Going forward, it is important to further corroborate each link in this model and delineate boundary conditions. It is also important to determine what aspects of this model have to do with BWT and what aspects do not. A broader and more challenging future direction is determining whether there are parallel psychological processes triggered by perceived disorder that collectively define a unique constellation of psychological phenomena.

## Conflict of Interest Statement

The author declares that the research was conducted in the absence of any commercial or financial relationships that could be construed as a potential conflict of interest.

## Footnotes

1 Across the selected studies, they control for various community- and individual-level variables such as urban area, neighborhood disadvantage, race, education, and income that may correlate with the dependent variables, yet perceived disorder remained a significant covariate.
